# “I am the Mother of the Boss here”: A Relational Biography of a Drug Dealer’s Mother

**DOI:** 10.1007/s10612-025-09854-z

**Published:** 2025-09-15

**Authors:** Verónica Zubillaga, Manuel Llorens

**Affiliations:** 1 Mellon Visiting Professor, Department of Latin American and Latino Studies, University of Illinois at Chicago, 601 S. Morgan St., 1525 UH, Chicago, IL 60607 USA; 2 Escuela de Psicología, Universidad Católica Andrés Bello, Urbanización Montalbán, Parroquia La Vega, Municipio Libertador, Apdo. 29068, Caracas, Distrito Capital, Venezuela

## Abstract

This article explores the rarely told story of the mother of a drug dealer and gang leader. Through the relational biography of Virginia and her son, we will grasp the importance of family networks and their role in exercising violence, giving rise to contexts of what we call “lethal reciprocity” in the neighborhood. Virginia´s story reveals the sinuosity of the exercise of power in her community through her filial bond with her son. Amid chronic vulnerability, her maternal position in the neighborhood allows her to obtain certain advantages by knowing how to manage her son’s capacity for violence, the basis for some of her son’s decisions and actions. At the same time, her position as a mother of this figure simultaneously places her in a position of vulnerability, of permanent risk in front of her son´s enemies, and victimization of her persona and loved ones. This tension, a dialectic between privilege and vulnerability, runs through her whole life experience.

## Introduction

“Because I am the mother of... as they say, of the boss here, of the one that everyone pays attention to [*todo el mundo le para bolas*].... they don´t mess with me because if I tell my son, my son stops it. This place eats you alive, girl, even the old, you know?” -Personal interview with Virginia on April 4, 2011. She was 49 years old.

“Well, because it has always been like that.... They are afraid of him, aren't they? Because Roberto [Virginia’s son] is not going to take it if they mess with me, and not only with me, but with the whole family. He´s in jail, but Roberto picks up a phone and blood will run everywhere. That's what I do, to avoid problems, I know that's how it is…” -Personal interview with Virginia on May 6, 2022. She was 60 years old.

These two quotes are taken from interviews carried out with Virginia more than ten years apart. Virginia is the mother of Roberto, a *malandro* [armed thug], who is the head of the drug distribution business in the poor neighborhood in Caracas, the capital of Venezuela, where we have been carrying out research since 2009. The first interview, carried out in 2011, was part of a broader research project that investigated the social and political processes surrounding the establishment of a ceasefire pact between two local gangs that had been engaged in armed confrontations for at least a decade and a half (Zubillaga et al. 2014). As the boss of one of the two gangs, Virginia’s son, Roberto, had been involved in the confrontations. Virginia also had a significant role, however, not only as his mother but also as a member of a local women’s group brokering the ceasefire. We spent a significant amount of time with her, including formally interviewing her, both individually and in the context of the many group interviews we carried out.

Since this initial research, we have kept in regular contact with this community for various purposes. In 2022, we returned specifically to interview Virginia in relation to our participation in Dennis Rodgers’ ERC-funded GANGS project.^1^ We endeavored to gather her life story and understand her experience of being the mother of the gang leader in her community. Given her persistent highlighting that she was “the mother of the boss”, we wondered how she navigated her son’s violent engagements and illicit activities, both now and throughout her past life? How did she handle her everyday relationships with her neighbors, given her link with this prominent figure? In what ways did her particular experience of motherhood shape her life and how did it allow her to grapple with challenges and difficulties? With these questions in mind, we carried out six biographical interview sessions with Virginia, which were also informed by the ethnographic research on drug trafficking in the neighborhood that we carried out between 2009 and 2012, and in 2014 and 2017.

As Virginia’s words reveal, a central motif in her biographical account is the privilege—albeit in tension with the vulnerability—of being the mother of the head of the local drug business. Likewise, a theme evident throughout her biography was the importance of family networks and their role in exercising violence, and in particular, how they often gave rise to a lethal economy of violent reciprocity in the neighborhood. These issues intertwined with issues of motherhood and agency, highlighting the ambiguities surrounding violence in contexts of extreme armed urban conflict. To this extent, this article can be said to respond to the call by Jensen and Rodgers (2022) regarding the need to better understand how violence and the dynamics associated with drug trafficking “fold” into the intimate lives of the individuals involved and the communities within which drug trafficking occurs, including especially women, so often ignored (see Gay 2005 and Rodgers 2024).

Through Virginia’s voice, we explore the understudied gendered experiences of motherhood in relation to the biography of a male gang leader. We aim to grasp the dialectic between privilege and vulnerability that runs through her life experience as a result of this position and connection, highlighting the intersection of gender, social disadvantage, and age in order to understand—from a structural and historical perspective—the concatenation of violence and survival strategies deployed by mother and son in the context of a Latin American city such as Caracas (see Crenshaw 1989; Adler de Lomnitz 1975; Auyero and Berti 2015). Despite the oil wealth of twentieth-century Venezuela, their stories are set against a backdrop of a profound and chronic neglect of the urban poor by the State. A persistent abandonment that has continued into the twenty-first century, despite the promises of social and political transformation associated with the so-called "Bolivarian revolution", that began in 1999 under the presidency of Hugo Chavez, and which defined itself as “socialist” and at "the service of the poorest" (Smilde et al. 2022). The life experience of Roberto—who has been in prison during the whole of our interactions with Virginia—further translates the carceral punitivism that has prevailed in Venezuela from 2011 onwards, and which made prison a more than likely fate for many young males from popular sectors of Caracas (Antillano et al. 2016; Zubillaga & Hanson 2018).

Virginia recounted her life in a manner that linked it intimately to her son’s. The preeminence of this bond in Virginia’s story resonates with the call for a more relational approach in social analyses (Bourdieu and Wacquant 1992; Moreno 1995; Mische 2011). The need to deploy a gendered relational approach became evident due to the preeminence of the maternal-filial link in Virginia’s self-presentation and narration of her story. She continuously repeated phrases such as "I am the mother of the boss here", for example, highlighting how her life experience is deeply marked by this filial relationship (see Moreno, 1995). Indeed, the vicissitudes of her son’s trajectory in many ways become her own, with her biography intimately constituted through the intersection of two generations, two gendered trajectories, and two life-worlds, as the personal stories of both Virginia and Roberto become interlinked plots that affect each other unceasingly throughout their life courses. The biography presented here is thus the account of Virginia’s life and that of her son as perceived and narrated by her. Her story allows us to understand both her trajectory, as well as that of a young man from a poor Caracas neighborhood and his involvement in drug trafficking.

From a subjective perspective, their interlinked stories reveal the specific suffering of intersectional violence in the domestic and public spheres, and its disparate effects on men and women (Scheper-Hughes and Bourgois 2004; Hume 2009; Hume and Wilding 2019; Allum 2024). Whether it is the accounts of sexual abuse experienced by Virginia in her stepfather’s home, Roberto’s experience of harassment by other men in his neighborhood, or his being shot in the stomach, these all reveal how these sufferings concatenate with others and how their effects spill far beyond a specific individual. As such, we show how the maternal filial bond between Virginia and Roberto organizes the meanings of violence and its use, as well as how, for Virginia, it clearly constitutes a form of social capital and privilege amidst the general marginalization characteristic of the local context. Although she is a victim of extreme structural violence, Virginia’s position as Roberto’s mother allows her to develop forms of agency and power to deal with adversity and obtain certain advantages in her neighborhood. Of course, we are dealing here with the paradoxes of a precarious agency (Santacruz 2019). The gang violence embodied by Roberto is both a cause of Virginia’s suffering but also the means through which she survives and navigates a violent context.

Through Virginia’s relational biography with her son, this article explores how family networks shape violence, creating contexts of what we call lethal reciprocity in the neighborhood. Virginia´s story reveals the ambiguities related to her filial bond with her son. Her maternal role grants her certain advantages as she navigates and influences her son’s capacity for violence, and also informs some of his decisions. Simultaneously, being his mother exposes her to risk, making her vulnerable to his enemies and subject to victimization. We term this tension the dialectic between privilege and vulnerability, and consider how it has conditioned her life. This article is structured into four sections that chronologically trace Virginia’s life story, from her late childhood to the time of our most recent biographical interviews in 2022. Central to her narrative are the experience of motherhood, the development of a sense of agency, and her relationship with her son, Roberto, and we examine how each stage conditions how she navigates and manages her son’s capacity for violence.

## Growing up at the Crossroads of Violence: The Experience of Structural and Sexual Violence

The story of Virginia’s childhood reveals how multiple expressions of violence overlap—including especially forms of structural and domestic violence—to shape women’s experience in Latin America’s poor, marginal urban neighborhoods, albeit often in a hidden manner (Hume, 2009). In particular, she began her story by narrating the difficulties of her childhood: “Mom worked every day cleaning houses here in La Sabana. But it wasn’t enough because there were five of us. Money was also a problem back then. There was money in the country, but for a person who cleaned, what she earned was not enough to eat. I had to leave. And that is how my life has been.” Virginia was forced to abandon her studies when she was nine years old to work on the street: “I didn’t make it past third grade. I had to work because there was always a lack of food, all the time, just like now. Of course, now it’s because of the country´s situation; at that time, it was because you were raising many children.” She also reported witnessing her father beating her mother: “He would beat my mom every day because he would ask my mom for food, and my mom would cook everything for us, and there was no food for him. My mom filed a report with the police about my dad, but nothing ever happened, it was one disaster after another.”

Virginia’s mother left her father for another man when she was twelve. Her mother’s new husband sexually abused her at the age of thirteen. Virginia experienced a form of double violence when her mother did not believe her, and she was left caught between the abuse from her stepfather and her mother’s disdain. It was then that she decided to leave her home, with, as she put it, “the first guy who turned up”: “So, look how my life was going… I said to myself I’d better not stay here. I’m not going to let my stepfather undress me. I told myself I’m leaving with the first guy who turns up… And that’s what I did! [The man who became] Roberto’s father came by, he had just left the army, and I left at once. I said: ‘Before this guy [her stepfather] fucks me, let someone else fuck me,’ and so I left with Roberto’s father [to be]. He took me to Anzoátegui and there we got a little piece of land.” Virginia was fifteen when Roberto was born, something that clearly brought her great happiness and fulfilment. Her suffering did not stop, however; on the contrary: “The guy [Roberto’s father] spent every day drinking *aguardiente* [alcohol]. I also worked cleaning houses in La Victoria. I would leave Roberto with a girl, a neighbor of mine, and she took care of him, and I paid her. In those days Venezuela was Venezuela. In the houses where I worked, they gave me boxes of diapers”. One day, however, Roberto’s father came home drunk, she continued: “Roberto’s stomach was aching, and he was crying. His father got mad at him and said he was going to beat him. Immediately after that, I left him and came back to Caracas with my little boy, where we are to this day!”.

Back in Caracas, Virginia recounted the anguish of being a single teenage mother, having to move from house to house, without a permanent home. Virginia finally found the house she still lives in today in the informal neighborhood of La Caracola.^2^ She was among the first wave of squatters who founded the neighborhood. Now settled in a house, it was necessary for Virginia to find a source of income to support her son. With no formal education, prostitution seemed the most feasible option open to her, all the more so as her local friendship networks included women engaged in this lifestyle and economic practice, called “*fichar*”. She told us about it during the following exchange:

- Virginia: I worked a lot as a whore, you know? For many years I worked as a whore and helped my family a lot.
- Verónica: And how did you get started in that trade?
- Virginia*: Oh, Mija!* With friends, you know. They took me to bars, where you start to get to know people. I worked as a *fichera* for many years. I started working in that job out of necessity. I was 17 years old. A friend of mine lent me a *cédula* [identity card] because she looked like me, she was of legal age, she was eighteen, and that was what I used to work with.
- Verónica: In bars? Or on the street?
- Virginia*:* In bars. Not in the street, in bars. I was afraid in the street. I worked from 9 p.m. until 5 o’clock in the morning, to raise Roberto. But it destroyed me. Every day I was *curda* [drunk] because I was working, I was *fichando*… And that life is tough, you know?

Research focusing on the strategies of disadvantaged women has frequently highlighted how prostitution can be a way for them to actively provide for their family. McCurn (2020), for example, describes how the intersection of gender, social disadvantage, and place of residence defines the economic survival strategies of poor urban women, allowing them, even at the cost of suffering and physical tribulations, to forge a particular agency and creativity amid economic disadvantage. This perspective helps us understand Virginia’s choices, but she also described to us how it was an ambivalent experience, on the one hand, because of the suffering and the shame of the stigma associated with the trade, but on the other, because it brought her great pride to be able to provide for Roberto, as well as for her younger sisters. At the same time, her relationship with him evolved as he grew up, as well as her role as a provider, as the next section details.

## Becoming the Mother of the Malandro

The beginning of the 1990s marked a period of deterioration in the living conditions and loss of hope in upward social mobility in Caracas (Briceño-León 1999). This context saw the expansion of criminal activity, with drug dealing becoming a critical source of income-generation for many poor, young urban youth, through which they not only sought to survive but also actively resist exclusion from conspicuous consumption (Pedrazzini and Sanchez 1992; Briceño Leon and Zubillaga 2002, for similar experiences in other contexts, see Bourgois 1995; Rodgers 2007).

From Virginia’s perspective, the rise of drug dealing was initially hugely problematic, as armed confrontations between rival gangs involved fighting over markets and turf became commonplace in her neighborhood: “There was a gang called *Los Parchis*, who were from a neighborhood further up. They started to come down to ours, and would hang out here. But all of a sudden, they started a fight, because they tried to steal drugs from Walter [the leader of the local La Caracola drug gang]. That’s when the fights started with those up there. And that led to other things when later, the ones from up there went and shot Martincito, who was Walter’s first cousin, which really stirred things up.” Walter, the head of the La Caracola drug business and brother of Sara, the woman who later became Roberto’s partner, is an essential character, whom we will return to later. However, at this point in Virginia (and Roberto’s) story it is more important to highlight that during his adolescence, Roberto began to experience systematic harassment and humiliation from other older adolescents in the neighborhood. Karina, his aunt, described it this way: “When my nephew was growing up, those older than him used to mess with him, they would beat him up. …There were problems among the boys here who wanted to subdue him, that he was a little faggot, that this, that, and the other. I think he got tired of so much abuse.”

As we know from the literature on biographical narratives, this kind of personal experience often leads to intense moral emotions, such as rage and impotence, which can be turning points in personal trajectories (Strauss, 2008; Katz 1988). The persistent harassment culminated in a critical incident whereby Roberto was shot when he was 16. His aunt described it as the beginning of his life as a *malandro*: “When he was shot, he told my sister while being taken away for surgery that if he survived, he was going to become a *malandro*. That they were not going to fuck him anymore…” From Virginia’s perspective, this was, indeed, the event that marked the transformation of her son´s lifestyle: “Roberto began his adventures when he was 16. I told you that Yesenia’s [his first girlfriend, with whom he had a daughter] cousin shot him. Well, he shot him, and Roberto told me these words in the hospital: ‘Now I’m going to become a badass, really, Mom, you’ll see’. Because they were playing. It was a carnival, they were playing, and the boy was in love with my daughter-in-law, and just for that reason he came and shot him.”

From that time onwards, Virginia claims that she was unable to control her son: “After he was shot, I took him to his father’s house in Zoata [in the interior of the country].^3^ But he came back again by his own means after a few weeks, and I couldn’t control him anymore. I couldn’t. When he came here, it was a fight, and a fight, and a fight, and I left La Caracola and went to Ciudad Bolivar for a while because I got tired, I got tired of fighting him; that’s when he got into it the most.” It was around then that Roberto began selling drugs to “get by”. He started in Walter’ gang, who ran the business in the neighborhood: “Walter was the boss of everyone here. He was the one who moved the kilos, the one who brought everything in. Once he had drugs stolen from him here, and he shot the guys. And I tell you, if he surprised anyone stealing from him, even family, he would kill them. Walter gave him [Roberto] a little bit to get by. He needed the money and asked Walter, and anyway, he was already hanging out with him. The first time he started selling, it was *basuco.*”^4^ Although Roberto was very young at the time—16 years old—he started moving up the echelons of the drugs trade. He also started going out with Walter’s sister, effectively becoming his brother-in-law. As Jensen and Rodgers (2022) have highlighted, family and neighborhood relationships often play a primordial role in the organization of illegal economies, and this relation became important in consolidating Roberto’s place in the local drugs trade, but also drew him, through the logic of reciprocity that engulfs family members, in extremely lethal tit-for-tat conflicts, known in Caracas as “*la Culebra*”,^5^ or “the Snake” (see Smilde, 2007; Zubillaga, 2008): “Walter got him into that horrifying world. And well, all these fights here began because they started killing Walter’s siblings. The cousins came and responded. They all wanted to have the cemetery to themselves. They have killed too many here, *chama*, too many: the nephews, the brothers, the cousins, the uncles, it’s crazy, you hear!”.

Walter was eventually killed by one of his enemies, and Roberto, as his brother-in-law, inherited the business: "What happened there, I am going to tell you honestly, is that Roberto got together with Sara [Walter’s sister and Roberto’s girlfriend at the time], and you know that Walter was the boss there. So when they killed Walter, and Roberto stayed there, he inherited the business.” Virginia explained how Roberto felt mesmerized by the ascendancy he produced, by being desired and sought after: “I used to say to Edgar [her husband at that time], ‘Don’t you see that Roberto wants to stay in Walter’s place? Because I saw that everyone was calling him. Everyone was watching him as if he were Maduro [alluding to Nicolás Maduro, the current president of Venezuela], and he liked that! He liked that, you hear? And that's why he is the way he is”.

Roberto rapidly expanded Walter’s old business: "Walter always sold by the kilo, but Roberto did so in bigger quantities: 15, 20, 30 kilos or more! He was a distributor. Crowds used to come, *mija*! And in big, expensive cars, you know the rich are more *messed up* [meaning addicted to drugs] than anyone. The people with money came and bought by the kilo. All that [the drugs] came directly from Colombia. I know the man, the one who brought it to him. That Colombian guy, he’s still alive! And he’s still involved in it. He would bring it to the barrio himself. Then *El Negro* [Roberto’s subordinate who stored the drugs] kept it, and Roberto would call the people he trusted who would buy it from him. That’s why Roberto met so many people. Many people would call him to ask if the merchandise had arrived, and they would come to pick it up. Roberto would ship it, put it here, there, wherever by the kilo”.

## The Dialectic of Privilege and Vulnerability

From the first time we met Virginia in 2009, it was clear that she was the mother of the boss of the gang. In her lively interventions during group interviews with the women, she repeatedly emphasized her identity as a mother, but not just any mother: she was the mother of the “*manda-más*” [the boss], insinuating her superiority in the hierarchy of the social space of the neighborhood. The women and the neighbors whom we talked with confirmed this position of ascendancy. At the same time, behind her back, they often commented on Virginia’s alcoholism, revealing the tensions among them.

In the everyday life conflicts in the barrio, where she and her family were involved and she felt threatened, Virginia menaced her neighbors with Roberto. In our group discussions, for instance, we were told of a time in 2011, when the La Caracola community faced the possibility that the garbage collection would cease due to new land invasions and the building of houses that impeded the transit of the collector trucks. Virginia warned she would bring her son to "*shoot everyone*." She made the threat concerning a situation that she perceived as an unsustainable abuse that endangered her family’s health.

Furthermore, after Roberto had become the head of the local drug business, Virginia engaged in public performances to display her power over her son. She was excited when recounting one encounter that took place in 2011: “José [a neighbor] said that my son had killed someone from Palito[a neighboring area]. And then I began trembling, I got very upset. I was coming from the market, and when I saw him [Roberto], I shouted at him and hit him, and he said ‘But Mom, I didn’t do it, I didn’t do it!’ I kept on shouting at him: ‘Tell me the truth!’, I was going crazy. Karina, Virginia’s sister, described this very public spectacle of her sister hitting her son, as highlighting “the respect” he owed her for being his mother: “My nephew, despite his age and as bad as he was supposed to be, let my sister hit him in front of everybody. The other day he was with a group of friends and she came down and slapped his face! And one of those thugs, one of his friends said: ‘And that old witch can hit you?’ My nephew answered: ‘That’s my mother, don’t mess with her, she’s my mother!’ That’s the respect that he has for her, you understand, independently of whatever he is, he said: ‘That’s my mother, and don’t ever mess with her’.”

Virginia’s maternal ascendancy over her son and her concomitant ability to have him be violent for her was something that played out through different spheres and contexts. For example, during an interview, Virginia recalled the periods of intense confrontation in the neighborhood, and the demands she made on her son then: " There was a moment, during a shooting when I was with my little daughter, and I said to Roberto: ‘Get the machine gun out, get it out!’ [laughing] Kill them before they kill us!”. At the same time, immediately after recalling this event, she sought to present herself as a mother with a positive influence over her son, who would appeal to his prudence and mercy in the exercise of violence, for example saying that: “I always told him: ‘You have to investigate well!’ For example, when my niece, his cousin, was killed, I told him that he could not kill people like crazy. I told him: ‘Check and see who did it, and with those who did it, make them pay, but no killing innocent people, OK? I was very strong with him regarding that issue…”.

Virginia also narrated how she would also intervene to defend her own family from Roberto’s fury when it came to drug debts: "Drugs are problems. The one who consumes has to pay because, one way or another, Roberto is also going to be charged. What Roberto didn’t do was fuck people over because they owed him, but he charged them, he charged them. I have a niece, Luisa, Karina’s daughter, and she is really ‘messed up’ [addicted to drugs]. Once she stole 500 g from Roberto, you hear? He wanted to kill her. He put a gun to her head, and I had to take it away from him. Sara, Roberto’s wife, and others grabbed her and beat her up instead, which was the lesser evil. I then told Roberto that I would never forgive him if he spilled his own blood. Because I don’t agree with that, right? How am I going to let my son shoot my niece? And Karina has always said she thanks me for that”.

The question “How can I let my son shoot my niece?” reveals Virginia’s belief in the importance of family ties, and how “blood” calls for loyalty. At the same time, it was also a source of major uneasiness in her life, and even if she often bragged about her power derived from being Roberto’s mother, the guilty feelings that derived from the complications associated with his activities also came to the fore on some occasions. This included an extraordinary exchange that we had with her during a group interview with the women of La Caracola, for which she arrived drunk and then asked to talk privately to one of our research team. During that conversation, she shared the burden of living with the weight of her son’s violent lifestyle and the knowledge that her words could have life-or-death consequences. It was extraordinary to hear her pain and guilt in this regard, as they were generally not evident. The fact that she could only express these feelings while drunk, outside the frames of the programmed interviews we had with her, evidence the difficulty of sustaining the conscious awareness of many of these contradictory feelings. It also suggests a dissociative coping style that is frequent in survivors of chronic violence (Moskovitz 2004; Llorens 2022).

Virginia’s position as Roberto’s mother also simultaneously places her in a position of vulnerability and permanent risk in relation to Roberto’s enemies, precisely due to the same moral logic of familial responsibility and lethal reciprocity. In particular, her narrative about her life highlighted how she was exposed to her son’s networks of animosity and threats. Virginia frequently told us how anxious she was during periods of armed confrontation, fearing that she would be targeted, and also detailed the experience of receiving death threats from Roberto´s enemies. She was also regularly warned to "go and buy a black suit" because they would kill her son. Nevertheless, her son’s reputation for vengefulness paradoxically also constituted, according to Virginia, a capital that allowed her to prevent aggressions against her own family: “Once, some guys from around here, from Portillo, saw me at the corner. They saw me there and said: ‘Look, there goes Roberto’s mother; let’s go fuck her up.’ They were overheard by somebody who worked with my niece, and he told her that he’d heard one of them say: ‘We’re going to hit Roberto's mother.’ But he then also heard another one say: ‘*Huevón*, don’t mess with that guy’s mom, he’s going to kill your whole family.’ But just the same, when I heard that, I stopped going out. I didn’t leave my neighborhood for about two months. But it’s a problem. Roberto once told me: ‘Oh, Mom, they’re not crazy. If they touch you, they’ll have to see everyone in their family die, from the youngest to the oldest, they know that.’ And I told him: ‘That’s what I don’t want. I have the years that God gave me to live, and no more, and you will not revive me by killing children and old people and everybody else, right?”.

Despite these misgivings, Virginia nevertheless continuously alluded to the “respect” earned by her son during our interviews. She, in particular, emphasized his moral code, arguing that this regulated his exercise of violence and what allowed him to forge a reputation of being “serious” and inspired respect in the community. As Bourgois (1995) has highlighted, respect is a fundamental identity capital in marginal neighborhoods such as La Caracola, and Virginia was adamant that “Roberto is very much loved and respected. People always say that Roberto is a serious guy, that Roberto is outstanding.” This respect transferred to Virginia, who would often explicitly remark on this: “Look, they respect me because I am Roberto’s mother, right? Some people tell me that it’s because nobody dares to tell the truth like me, ta, ta, ta. But they have also told me: ‘Who dares to mess with you, Virginia? Everyone is afraid of Roberto, because, let´s be straight, that´s how it is’.” This was the case even though Roberto was in prison. As Virginia put it, she can call him, and “he picks up the phone and blood runs everywhere. Often I don’t even have to do that to avoid problems; everyone know that’s how it is…”. The threat of her son’s potentially devastating responses constitutes one of the key resources available to Virginia to actively negotiate the limitations imposed on her and her family in a broader context of extreme violence and disadvantage (Hume and Wilding 2019: 5).

## Carceral Experiences

Roberto was arrested on March 9, 2011, when he was 34 years old. According to Virginia and Karina, he was denounced to the police out of “envy”. It is possible, however, that the scale of his drug dealing and his flamboyant lifestyle, as well as his growing notoriousness, had made him highly visible to the authorities. At any rate, prison was a predictable potential element of his trajectory, something that both Virginia and Roberto knew. As she explained: “Roberto is not an idiot. He knew that someday he was going to get caught, and so he saved his money, and he can still pay for everything.” At the same time, however, when Virginia spoke about her life after Roberto’s imprisonment, her discourse began to include a lot of references to her “suffering”. In July 2011, after two years of the implementation of a policy of systematic mass incarceration of poor urban youth by the authorities, Venezuelan prisons were overcrowded and suffered a series of riots and armed clashes among prison gangs and between prison gangs and custodians (see Antillano et al. 2016; Zubillaga and Hanson 2018). The 2012 crisis in El Rodeo prison, when a group of prisoners armed with firearms took another group hostage, demanding better living conditions, kept the whole country on edge. Roberto was in that prison, and so the crisis directly affected Virginia. She participated in demonstrations and protests by the families of inmates, including especially mothers, which often ended in pitched battles outside the prison between police officers and the relatives of detainees.

By the time we conducted our second round of biographical interviews with Virginia in 2022, Roberto had been in prison for eleven years. He had been in at least five different prisons. Despite all the appeals filed by his defense team, the Supreme Court of Justice confirmed his 15-year prison sentence in 2016. In the years following her son’s imprisonment, we saw the emotional and physical impact that the anxiety caused by his absence had on Virginia, including, in particular, increasing her already heavy drinking. During several of our conversations, Virginia showed us photos of Roberto in prison, commenting on the changes in his body: in some photos, he was thin; in others, he was “fatter”. The passage of time and confinement were visible: “Did you see Roberto’s white hair? I can see it; he is getting old”. She kept repeating: “I miss him, I miss him”. Virginia communicated every day with Roberto via their cell phones. This constant communication allowed her to continue to influence the actions of her son, who continued to give orders and control local drug dealing from the inside that were carried outside, in the neighborhood: “He is in prison, but Roberto picks up a phone just the same, and blood will flow here.” At the same time, Virginia administrated her power by managing the information she gave Roberto: “For example, when this problem occurred [a crack-addicted neighbor who was causing disturbances], I didn´t want to say anything to Roberto, because he would have just killed him, but someone always calls Roberto and tells him what happens, and so Roberto then called me. Yesterday! So I told him a lie. I told him: ‘No, *Papi* [Daddy], everything is fine here’, but I said it to avoid problems. I don’t want there to be any more deaths.”

Without being a prison boss in the social structure that rules inside Venezuelan prisons (Antillano 2023), Roberto nevertheless occupies a preeminent position that has allowed him to secure a cell that looks like an apartment. In the photos she showed us, Virginia proudly pointed out his big bed, the ceramic tiles he has had installed on the floor, his television, and other furniture and fittings. Amid the tragedy of not having her son close to her, and the complications she has to endure on the rare occasions she can visit him in prison, the resources, both material and immaterial, that Roberto has accumulated, and which are visible both in his luxurious prison conditions as well as her being respected in La Caracola, allow her moments of joy and pride. Moreover, they continued to celebrate important dates such as birthdays, Christmas, and most importantly, Mother’s Day, together, albeit in the prison, as she described to us: “It’s ugly in there, but nevertheless, we are able to have our Mother’s Days and everything… Those days are great. You go there and have a lot of fun. There were even *mariachis* last time!”, she told us, showing photos of the Mother´s Day party and of the mariachis. Showing us some other photos, she added, “this is in *La Pica* prison, where we celebrated Roberto’s birthday, my birthday, Karina’s birthday, and everything”. Despite her enthusiasm, there was an element of ambiguity about such visits, however, as came out during our last interview session with Virginia, when she told us that Roberto did not want her to visit him in prison anymore, because it implied lots of waiting, distress, and other conmplications for her. Instead, she explained, he had told her: “No, Mom, I’m going to get out soon, stay calm”, before concluding wistfully: “I still wait every day for my son to come out of there. I have hope, *mija*”.

## Final Comments

The biography of Virginia highlights starkly the chronic experience of disadvantage experienced by many who live in poor neighborhoods in a city like Caracas due to drug dealing and criminal violence. Yet it also shows how the violence of individuals involved in such activities, such as Roberto, can be extremely ambiguous, clearly causing much suffering in La Caracola and beyond, but also sometimes benefiting his mother and others connected to him in the neighborhood, although it can also sometimes constitute a major disadvantage too. Thus, this narrative contributes to our evolving comprehension of urban and armed violence in Latin America through a gendered and relational lens, considering the perspectives of mothers of gang members from impoverished neighborhoods. It underscores not only their social hardships but also the micropolitical survival strategies that their link to gangs enables (Auyero and Kilanski 2015; Zubillaga et al. 2019).

In particular, by presenting Virginia’s biography as a relational, we seek to contribute to the understanding of the complexities of women’s responses to extreme violence by focusing on the lived local repertoire of actions associated with the particular contexts and webs of associations within which they live. Virginia’s story is the rarely told story of the mother of a drug dealer and gang leader, and by focusing on this relationship, it adds complexity to the literature focused on the intricacies and ambiguities of women’s agency and their role in the reproduction of everyday violence, something that is often overlooked in the literature, which usually tends to see women as the passive recipients of drug and gang violence (Allum 2024).

Several feminist anthropologists have called attention to the way women can strategically "use" their role as mothers to gain power in public arenas and access services (Martin 1990; Rodriguez 1994). In particular, Martin (1990) has described how the respect culturally associated with the figure of the self-sacrificing mother figure within Latin America encouraged women to participate in political activities under the banner of motherhood in Mexico. Similarly, using predominant gendered scripts to underscore her position as “Roberto’s mother”, Virginia frequently places herself in a position of social ascendancy within the neighborhood. Virginia’s discourse promoting her identity as the “*malandro*’s mother” allows her to strategically emphasize the weight of her power derived from the filial bond. Her position and assertion of her relation constitute a form of capital and survival strategy that helps to guarantee her and her family’s security using the threat of her son’s violence in a space where she would otherwise be helpless to protect herself. Likewise, the threat of her son’s violence is a resource to settle conflicts linked to the precariousness of rights and services in the neighborhood’s daily life.

But it is a double-edged sword of a resource. As Robert Gay famously portrayed in Lucía (2005), his now classical biography of the female partner of drug traffickers in a Rio de Janeiro favela, although she achieves a lifestyle of conspicuous consumption through her relationships with men linked to drug trafficking, she is also subjected to their violence, both directly, being shot in the leg by her partner in a jealous episode, and indirectly, suffering community opprobrium. Virginia, like Lucia, is also subjected to suffering caused by the accumulation of social disadvantages due to her association with Roberto, but unlike Lucia, she also asserts a unique and contradictory ascendancy over her son and his exercise of armed violence due to her position as his mother that also provides her with definite recompenses.

In particular, her life trajectory shows the ways that she exercises power and agency through her filial bond to Roberto, and uses her son’s capacity for violence to her advantage. Her story thus reveals the sinuosity of the exercise of power in her community, and how it goes much further than just gang members and other visibly violent actors. This is important because in contrast to the experiences of women depicted in ethnographies of violence where *silence* is one of the main strategies of survival in relation to men´s violence (Hume 2009; Hume and Wilding 2019), Virginia *shouts* at her son, “the boss”, in public. Even if elements of her s life very much conform to gendered scripts—she was, for example, sexually abused in her youth by her stepfather, something which led her to leave home with “the first man that passed by”, whom she subsequently left due to his violence—she also acquired social power as a mother through her son’s violent ascendancy in the community. As such her life story highlights deep tensions, a pervasive dialectic between privilege and vulnerability, whereby Virginia navigates very difficult circumstances of powerlessness due to broader social and gendered inequalities, confronting difficulties with a strong and determined will, yet, at the same time, doing so based on these same social and gendered inequalities through taking advantage of her particular positionality as the mother of the powerful man in her neighborhood.

Virginia’s story also shows how she makes efforts to present herself as being guided by a moral persona that she also projects onto her son amid the many violent and illegal actions in which her family is implicated. She seems to argue that her actions are efforts to contain and direct violence in “fair” ways. But her arguments tend to minimize the brutality of much of what is happening around her, as well as her own disposition to resort to violence whenever she feels threatened or confronted. For example, explaining her niece’s difficulties—and ultimate death—as being due to her being addicted, without considering that the reason that she was addicted due to the drugs her son provided. We could therefore describe Virginia’s moral reasoning as oscillating between arguing for the use of strength as self-defense—such as hitting her son to try to control him, or threatening her neighbors with her son’s violence—and to enabling her to care for others, for example when she describes her efforts to moderate his violence by alluding to blood ties or withholding the “whole truth” to avoid conflicts in the community. In this way, her own violence and guilty feelings, as well as the contradictions implied in these options, are often downplayed to preserve a positive view of herself and her son’s decisions.

Virginia’s life is inseparable from that of her son and her position within the social dynamics of her neighborhood. Traditional biographies often frame individuals in relation to their specific challenges, opportunities, obstacles, and epiphanies, eventually contextualized by historical and social relations. However, such approaches frequently underemphasize the profound—and continuous—impact that certain social relations can have. What one might term “biographical individualism” falls short in capturing the life trajectory of a poor, marginal woman like Virginia in urban Latin America, for whom family ties are central in the organization of her life (see also Adler de Lomnitz 1975). In this article, we explicitly adopted a relational biographical framework to make sense of her experiences and place center-stage the significance of her identity as “the mother of the boss”, highlighting how her maternal role shaped her struggles and her position in the neighborhood. Virginia’s story can in the final analysis only be fully comprehended by examining the intimacy of her filial bond, which profoundly influenced her life trajectory. Following Moreno (1995), we can say that this bond is not only important but essential, as Virginia understands herself, evaluates her self-worth, interprets the world, and enacts her agency continuously through this fundamental relationship.

Finally, in recounting her relational narrative Virginia is incessantly arguing and justifying how, amidst the extreme difficulties they have encountered, her choices have not been easy, and have been both facilitated and complicated by her relationship to her son, Roberto. There is, in other words, an effort in her discourse to highlight a certain “tragic honorability” in the midst of a life marked by both abandonment and intimate connection to violence. Yet it is important to note that Virginia does not portray herself as a victim. Even if her privilege as “the mother of the boss” frequently exposes her to vulnerabilities and helps perpetuate a seemingly never-ending cycle of violence, it is also the means through which she juggles adversity and takes advantage of the unique social power that this maternal bond provides her with. Even if this is something that is not given to all in similar circumstances, it highlights the need to think about women’s lived experiences of violence in poor urban neighborhoods such as La Caracola in a more nuanced manner that takes their specific local relational positionality into account, rather than thinking about them through generic categories.

## Footnotes

1 More specifically, our endeavors were part of the “Gangsters” sub-project, which involved the collection of individual biographies of gang members and other individuals linked to gang members from multiple contexts around the world. For more details, see the Introduction to this special issue.

2 The community’s name has been changed. La Caracola developed as an urban neighborhood of self-built homes starting in the late 1930s, and, like other working-class settlements in Caracas, increased its urban density in the 1950s. It is situated near Miraflores, the presidential palace, and has a long history of drug trafficking. In the 1990s and mid-2000s, it faced ongoing gang violence and mass killings. It is also known for a 2007 ceasefire pact between women and drug traffickers (see Zubillaga, Llorens & Souto 2014).

3 Roberto's father partnered with another woman and had a daughter after he separated from Virginia. Although she brought Roberto to live with him when he was 16, she says that he never took care of Roberto before, and has not taken any interest since Roberto left him after living with him for a few weeks.

4 In Venezuela, *basuco* refers to the mixture of cocaine base paste with other drugs, which is usually smoked.

5 The *culebra* can be understood as a system of exchange among men living in neighboring urban barrios, initiated by an offense that demands a response under the threat of the recipient's unsustainable degradation, with its extreme outcome being death (see Zubillaga 2008).

## References

[CR1] Adler de Lomnitz, L. (1975). Cómo Sobreviven los Marginados. México D.F.: Siglo Veintiuno Editores.

[CR2] Allum, F. (2024). Women of the Mafia. Cornell University Press.

[CR34] Antillano, A. (2023). Galaxia Prisión: Cómo la cárcel remodela la vida de las clasespopulares en Venezuela. Prisiones. Revista electrónica del Centro de Estudiosde Ejecución Penal, 2(3): 29–46.

[CR3] Antillano, A., Pojomovsky, I., Zubillaga, V., Sepúlveda, C., & Hanson, R. (2016). The Venezuelan prison: From neoliberalism to the Bolivarian revolution. Crime, Law and Social Change, 65: 195-211.

[CR4] Auyero, J., & Berti, M.. (2015). In Harm’s Way: The Dynamics of Urban Violence. Princeton University Press.

[CR5] Auyero, J., & Kilanski, K. (2015). From “making toast” to “splitting apples”: Dissecting “care” in the midst of chronic violence. Theory and Society, 44(5): 393-414.

[CR6] Bourdieu, P., & Wacquant, L. (1992). An Invitation to Reflexive Sociology. Chicago: University of Chicago Press.

[CR7] Bourgois, P. (1995). In Search of Respect: Selling Crack in El Barrio. New York: Cambridge University Press.

[CR8] Briceño-Leon, R. (1999). Violencia y Desesperanza. Nueva Sociedad, 164: 122-132.

[CR9] Briceño-Leon, R., & Zubillaga, V. (2002). Violence and Globalisation in Latin America. Current Sociology, (50)1: 19-37.

[CR10] Crenshaw, K. (1989). Demarginalizing the Intersection of Race and Sex: A Black Feminist Critique of Antidiscrimination Doctrine, Feminist Theory and Antiracist Politics. University of Chicago Legal Forum, 1: 139- 167.

[CR11] Gay, R. (2005). Lucía. Testimonies of a Brazilian Drug Dealer’s Woman. Philadelphia: Temple University Press.

[CR12] Hume, M., & Wilding, P. (2019). Beyond agency and passivity: Situating a gendered articulation of urban violence in Brazil and El Salvador. Urban Studies, 57(2): 249–266.

[CR13] Hume, M. (2009). The Politics of Violence: Gender, Conflict and Community in El Salvador. Malden, MA, and Oxford: Wiley-Blackwell.

[CR14] Jensen, S., & Rodgers, D. (2022). The intimacies of drug dealing: Narcotics, kinship and embeddedness in Nicaragua and South Africa. Third World Quarterly, 43(11): 2618-2636.

[CR15] Katz, J. (1988). Seductions of Crime. New York: Basic Books.

[CR16] Llorens, M. (2022). Civil society responses to violence in Venezuela: Anesthesia, revenge and empathy. In Smilde, D., Zubillaga, V. & Hanson, R. (Eds.). The Paradox of Violence in Venezuela: Revolution, Crime and Policing during Chavismo (pp. 211-231). Pittsburgh: University of Pittsburgh Press.

[CR17] Martin, J. (1990). Motherhood and power: The production of a women's culture of politics in a Mexican community. American Ethnologist 17(3): 470–490.

[CR18] McCurn, A. (2020). Surviving the grind: How young black women negotiate physical and emotional labor in urban space. Sociological Spectrum, 40(4), 227–246.

[CR19] Mische, A. (2011). Relational sociology, culture and agency. In Scott, J., & Carrington, P. (Eds.), Sage Handbook of Social Network Analysis (pp. 80–98). London: Sage.

[CR20] Moreno, A. (1995). La Familia Popular Venezolana. Caracas: Centro Gumilla y Centro de Investigaciones Populares.

[CR21] Moskovitz, A. (2004). Dissociation and violence: A review of the literature. Trauma, Violence & Abuse, 5(1): 21-46. 10.1177/1524838003259321 15006295

[CR22] Pedrazzini, Y., & Sánchez, M. (1992). Malandros, Bandas y Niños de la Calle. Cultura de la urgencia en la Metrópoli Latinoamericana. Valencia-Caracas: Vadell Hermanos Editores.

[CR23] Rodgers, D. (2007). Slum wars of the 21st Century: The new geography of conflict in Central America. Development and Change, 40(5): 949-976.

[CR24] Rodgers, D. (2024). Soraya, la Reina del Sur in Nicaragua. In Auyero, J. (Ed). Portraits of persistence (pp. 21-38). Austin: University of Texas Press.

[CR33] Rodriguez, L. (1994). Barrio Women: Between the urban and the feminist movement. Latin American Perspectives, 21(3): 32–48.

[CR25] Santacruz, M. (2019). Mujeres en pandillas salvadoreñas y las paradojas de una agencia precaria. Papeles del CEIC, 2019(1): 1-20.

[CR35] Scheper-Hughes, N. & Bourgois, P. (eds.) (2004). Violence in War and Peace: An Anthology. London: Basil Blackwell

[CR26] Smilde, D. (2007). Reason to Believe: Cultural Agency in Latin American Evangelicalism. Berkeley: University of California Press.

[CR27] Smilde, D., Zubillaga, V., & Hanson, R. (Eds). (2022). The Paradox of Violence in Venezuela. Pittsburgh: University of Pittsburgh Press.

[CR28] Strauss, A. (2008 [1959]). Mirrors and Masks: The Search for Identity, New Brunswick: Transaction Publishers.

[CR29] Zubillaga, V. (2008). La culebra: Una mirada etnográfica a la trama de antagonismo masculino entre jóvenes de vida violenta en Caracas, AKADEMOS, 9(2):179-207.

[CR30] Zubillaga, V., & Hanson, R. (2018). Del punitivismo carcelario a la matanza sistemática: El avance de los operativos militarizados en la era post-Chávez. REVISTA M: Estudos sobre a Morte, os Mortos e o Morrer, 3(5): 32-52.

[CR31] Zubillaga V., Llorens. M., & Souto, J. (2014). Violencia Armada y Acuerdos Comunitarios de Convivencia: una larga marcha por la paz. Caracas: Editorial Equinoccio & Universidad Simón Bolívar.

[CR32] Zubillaga, V., Llorens, M. & Souto, J. (2019). Micropolitics in a Caracas barrio: the political survival strategies of mothers in a context of armed violence. Latin American Research Review, 54(2): 429–443.

